# Beam profile assessment in spectral CT scanners

**DOI:** 10.1002/acm2.12260

**Published:** 2018-02-07

**Authors:** Marzieh Anjomrouz, Muhammad Shamshad, Raj K. Panta, Lieza Vanden Broeke, Nanette Schleich, Ali Atharifard, Raja Aamir, Srinidhi Bheesette, Michael F. Walsh, Brian P. Goulter, Stephen T. Bell, Christopher J. Bateman, Anthony P. H. Butler, Philip H. Butler

**Affiliations:** ^1^ Department of Radiology University of Otago Christchurch New Zealand; ^2^ MARS Bioimaging Ltd Christchurch New Zealand; ^3^ Department of Physics and Astronomy University of Canterbury Christchurch New Zealand; ^4^ Department of Radiation Therapy University of Otago Wellington New Zealand; ^5^ European Organization for Nuclear Research (CERN) Geneva Switzerland

**Keywords:** beam profile, geometric calibration, Medipix detector, spectral CT, x‐ray source model

## Abstract

In this paper, we present a method that uses a combination of experimental and modeled data to assess properties of x‐ray beam measured using a small‐animal spectral scanner. The spatial properties of the beam profile are characterized by beam profile shape, the angular offset along the rotational axis, and the photon count difference between experimental and modeled data at the central beam axis. Temporal stability of the beam profile is assessed by measuring intra‐ and interscan count variations. The beam profile assessment method was evaluated on several spectral CT scanners equipped with Medipix3RX‐based detectors. On a well‐calibrated spectral CT scanner, we measured an integral count error of 0.5%, intrascan count variation of 0.1%, and an interscan count variation of less than 1%. The angular offset of the beam center ranged from 0.8° to 1.6° for the studied spectral CT scanners. We also demonstrate the capability of this method to identify poor performance of the system through analyzing the deviation of the experimental beam profile from the model. This technique can, therefore, aid in monitoring the system performance to obtain a robust spectral CT; providing the reliable quantitative images. Furthermore, the accurate offset parameters of a spectral scanner provided by this method allow us to incorporate a more realistic form of the photon distribution in the polychromatic‐based image reconstruction models. Both improvements of the reliability of the system and accuracy of the volume reconstruction result in a better discrimination and quantification of the imaged materials.

## 1 INTRODUCTION

A broad definition of the beam profile analysis encompasses all beam properties, such as spatial, temporal and spectral characteristics, power, and propagation. The characterization of a beam is specific to the type of beam, which could be monochromatic 1, 2, 3 or polychromatic.^4^ For a laser beam, parameters such as alignment, focus spot size, and beam uniformity are typically analyzed to optimize laser performance.^1^, ^2^ Characterizing the beamlines is also essential in particle accelerators for them to be operated with optimal output.^3^ The polychromaticity of the x‐ray beams used in the computed tomography (CT) scanners necessitates the accurate modeling of beam profile in these machines. CT scanners are used for diagnostic imaging (kilovoltage range) and image‐guided radiotherapy (megavoltage range). Many methods have been published for beam profile measurements of such systems. Among them, the work published by Malts et al. can be referred to. They presented a method of characterizing the spatial variation in the intensity and energy of the incident beam in diagnostic and treatment cone beam CT.^4^

Beam profile characterization is also a prerequisite to optimize performance of spectral CT scanners operating on the basis of photon‐counting detectors. The optimal performance of the spectral CT scanner is achievable when the energy and position of the incident photon are measured accurately.^5^ Beam profile assessment methods examining various properties of the beam profile can be used to identify the parameters that prevent accurate measurement of photon energy and position. The properties of the beam such as photon intensity and its angular distribution not only needs to be characterized at initial installation, but beam profiles also need to be regularly assessed for identifying the distortion caused by either deterioration of the x‐ray tube performance during its lifetime or instabilities of other scanner components. In this study, aforementioned properties are characterized for the beam profiles measured using MARS small‐animal spectral CT manufactured by MARS Bioimaging Ltd., New Zealand.

The MARS spectral CT scanners use Medipix photon counting detectors to provide 3D tomographic images with both high spatial and high spectral resolution. The energy resolved information enables simultaneous discrimination and quantification of different materials based on their spectral signatures.^6^, ^7^ The spectral imaging allows the extraction of functional and anatomical features of the tissues via tracing biomarkers and pharmaceuticals in a low dose and noninvasive way.^8^, ^9^ MARS imaging has been used in various preclinical applications such as characterizing the composition of excised vulnerable atherosclerosis plaques in arteries,^10^ functional imaging of arthritic cartilage,^11^ and targeting cancerous cells using nanoparticles.^12^

The x‐ray tube used in the spectral CT provides a cone shape photon distribution which typically varies over the imaging field.^4^ Furthermore, x‐ray tube manufacturing and alignment variation of the beam direction with detector plane in a spectral CT scanner also makes the photon beam profile specific to that system. For instance, relative geometric offsets due to tube anode orientation may spatially shift the recorded beam profile. To identify such a variation, the beam profile of each spectral CT scanner needs to be characterized. The information obtained from beam profile characterization can then be used to calibrate the image reconstruction models. Providing the more realistic form of the photon distribution to the forward model allows better image reconstruction, and as a consequence better material identification and quantification. Performing spectral reconstruction with an inaccurate characterization of the x‐ray beam has the potential to cause significant material misspecification.^13^

Reconstruction problems can also arise when random fluctuations occur in the beam profile due to instability of CT scanner components, such as the x‐ray tube and detector. Fluctuations in the beam profile are more likely when spectral data are acquired over a long exposure time. Relatively long exposure time is required because photon counting detectors can optimally operate at low photon flux.^14^ The use of low photon flux ensures maintaining spatial and spectral fidelity of the images in two aspects.^5^, ^15^ Firstly, the small pixel size of the photon counting detectors such as Medipix3RX favors the use of x‐ray tubes with small focal spot sizes (e.g., 50 μm) to maximize spatial resolution.^5^, ^14^, ^16^ Striking the smaller area of the anode target by electrons, in turn, generates a lower photon flux.^14^, ^17^ Secondly, due to limited pulse resolution time of such detectors, the energy information of a high flux beam cannot be resolved correctly. The energy of coincident photons is accumulated and registered at a higher energy of each initial photon. This pulse pile‐up effect results in the loss of spectral information.^16^, ^18^ To minimize the occurrence of this effect, incident photon flux needs to be reduced.

Acquiring data with longer exposure time, while using the low photon flux, provides sufficient counts; resulting in a higher signal to noise ratio in reconstructed images. However, the detector performance may degrade due to increasing ASIC temperature and as a consequence, charge loss occurs due to detector polarization during long acquisition time. Therefore, the beam profile stability needs to be monitored in such a system to ensure that there is no count drift during imaging. In response to this need, we have developed a beam profile assessment and characterization method. The method enables quantification of the temporal and spatial properties of beam profiles and assessment through comparison with modeled beam profiles.

In this paper, we introduce a parametrized semi‐analytic source model and the experimental requirements. We then explain the procedure of developing the beam profile assessment method, and present method evaluation results obtained from one well‐calibrated and two poorly calibrated MARS spectral CTs.

## 2 MATERIALS AND METHODS

**Figure 1:**
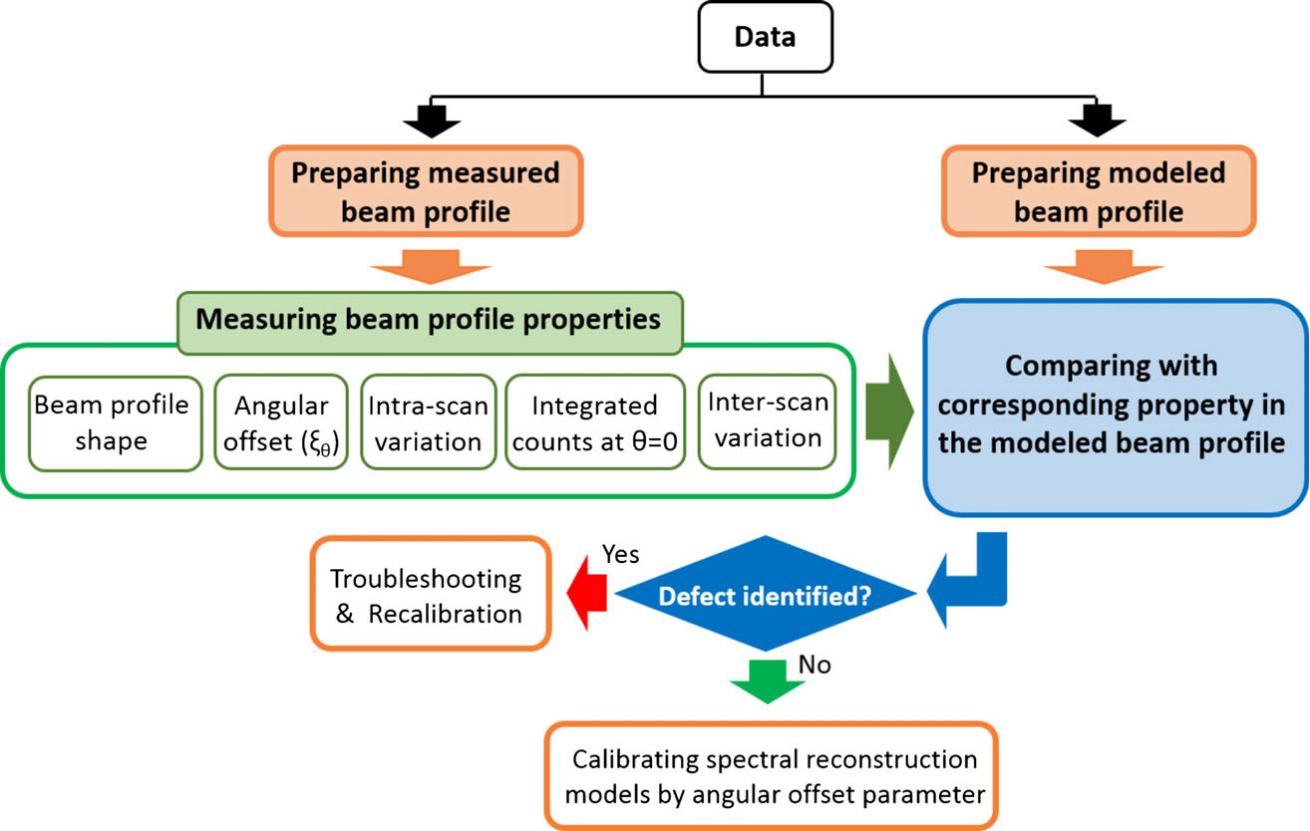
An overview of beam profile assessment method.

A workflow diagram of the beam profile assessment method is depicted in Fig. 1. An experimental beam profile is provided to the method. Then a modeled beam profile is prepared from a semi‐analytic source model based on the equivalent spatial parameters of the measurement. Measured and modeled data are then preprocessed to reach the same level of conformity to be comparable with each other. In the next step, several properties of the beam profile are measured. In the comparison step, the measured properties are compared with the modeled beam profile. If a significant discrepancy is identified, it indicates potential issues with calibration or components of the systems.

### 2.A Modeling the beam profile

**Figure 2:**
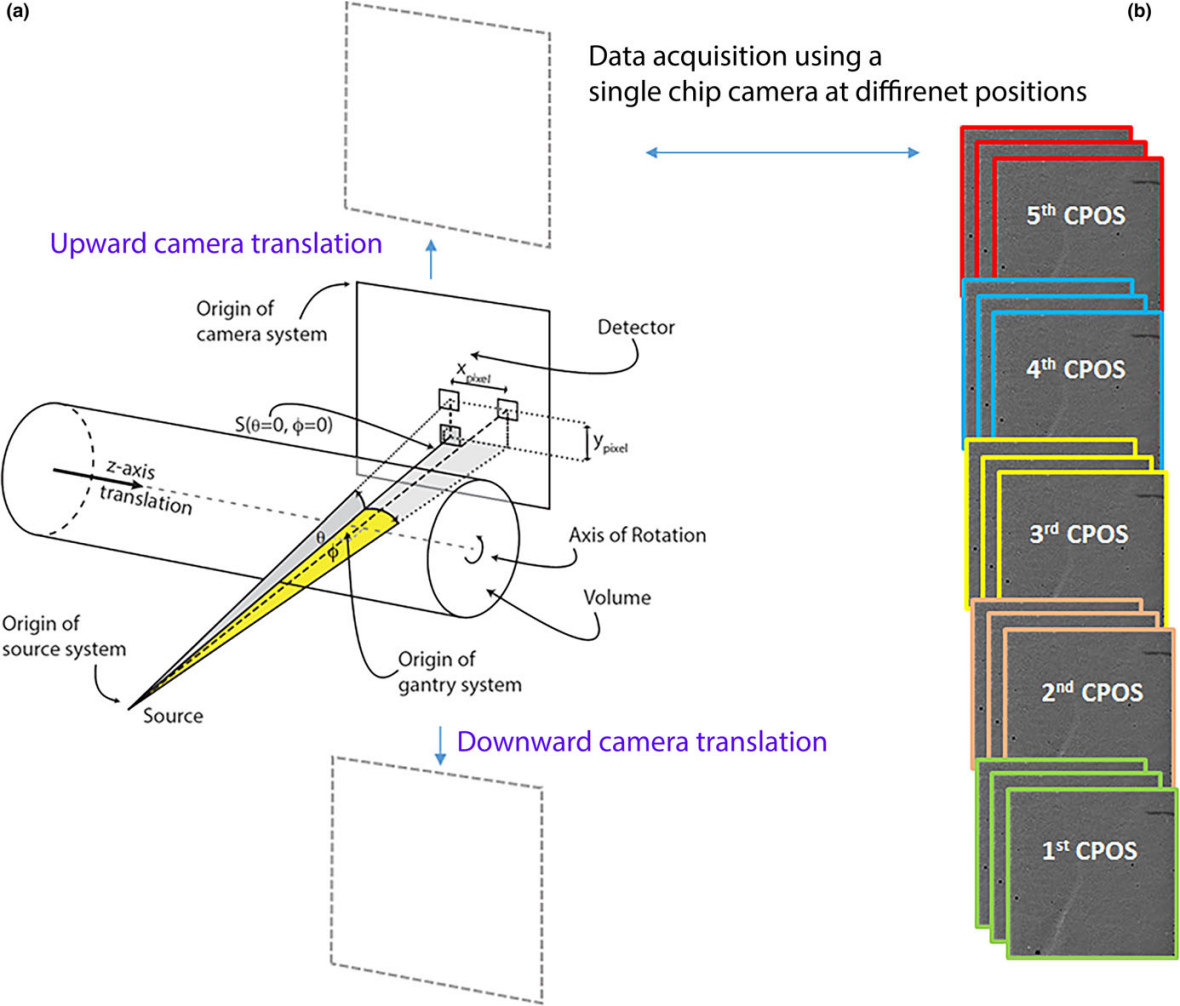
A schematic diagram of the components of MARS spectral CT with a single‐chip camera alongside the frame sequence acquired at each camera position (CPOS). (a) S shows the position of x‐ray source with respect to the detector plane that both rotate simultaneously around the object volume. The single‐chip camera is translated along the vertical axis. In this diagram, photon distribution along a solid angle in both θ and φ directions are also demonstrated. (b) A series of flat‐field images were acquired at each CPOS using a CZT‐Medipix3RX in a typical scan.

The beam profile assessment technique requires the use of an x‐ray source model that describes the spatial variation in the x‐ray beam away from the central axis. For this purpose, we have utilized a parameterized semi‐analytic source model fitted to the x‐ray tube with a 50 μm focal spot and 20° anode angle.^19^ The general formula of this source model, $S_{\mathrm{EV}}^{\theta \varphi }$, is presented by eq. (1).

(1) $$S_{\mathrm{EV}}^{\theta \varphi }=S_{\mathrm{EV}}^{00}[1+A\left(\varphi +\xi_{\varphi }\right)+B{\left(\varphi +\xi_{\varphi }\right)}^{2}+C{\left(\theta +\xi_{\theta }\right)}^{2}$$

where, $S_{\mathrm{EV}}^{\theta \varphi }$ provides the spectral components of the x‐ray spectra as a function of energy, *E*, tube voltage, *V*, and angular distribution of θ and φ. θ is the camera translation which is along the scanner rotational axis and φ expresses the anode–cathode direction that is orthogonal to the rotational axis and the beam direction as demonstrated in Fig. 2. *S* ^00^ provides the x‐ray spectrum at the beam center for a given tube voltage *A*,* B*, and *C* are coefficients, which depend on x‐ray energy (keV) and tube voltage (kVp). $\xi_{\theta }$ and $\xi_{\varphi }$ represent the beam offsets along θ and φ with respect to the center. This source model currently can be used for the x‐ray tubes with the voltage range of 30–120 kVp, and angular photon distribution within θ = ± 17 and φ = ± 5.5°. Further details can be found in Ref. [20, 21].

**Figure 3:**
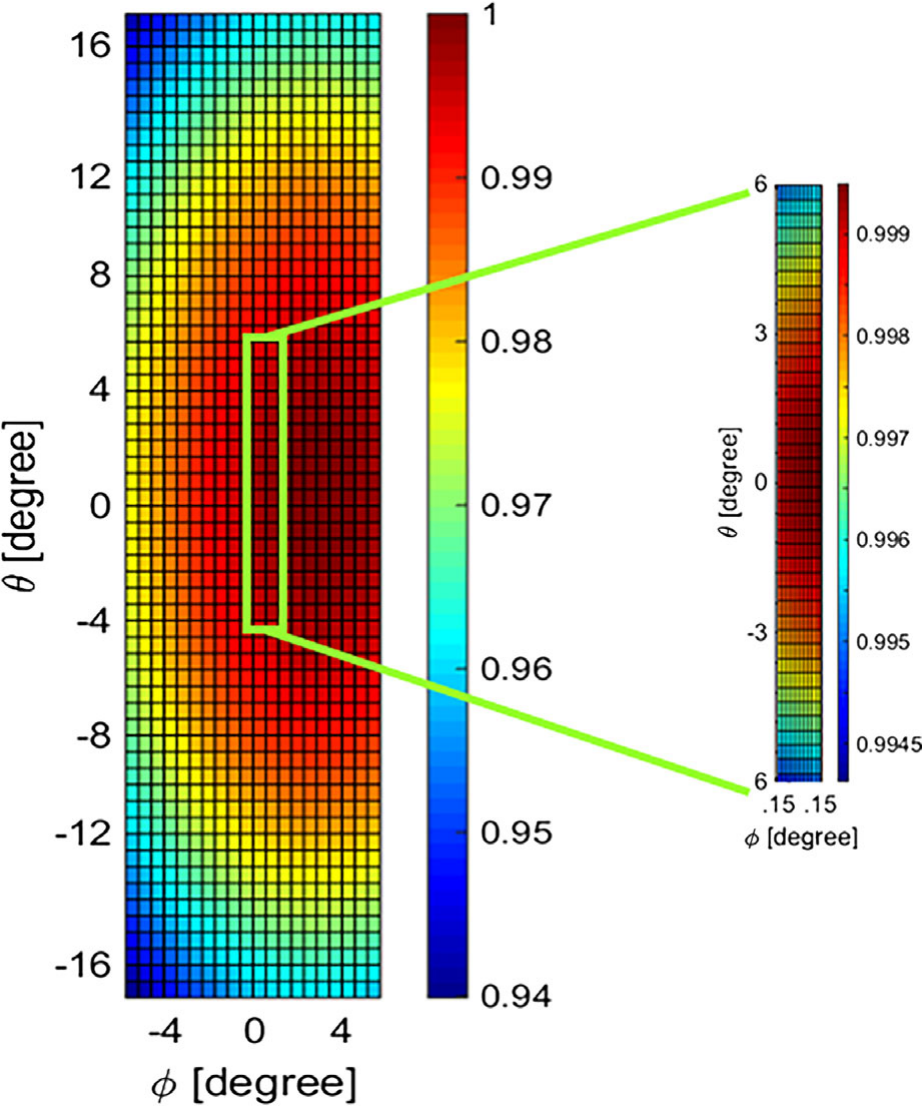
Normalized integrated count distribution of the source model. The magnified region identifies the portion of the beam targeted for the experiment.

To obtain a modeled beam profile, the first step is to extract a spatial photon distribution from the source model based on the tube voltage, filtration, and geometric features of the scanned data. The magnified beam profile shown in Fig. 3 is an example of a modeled photon distribution in a typical field of view fitted to scan a small object size like mouse. It should be noted that the x‐ray photon distribution across the rotational axis (θ **)** is analyzed in this study and the count variation along φ is assumed to be negligible (i.e., 0.06% in a typical field of view).

The second step is to correct the source model output for the factors that modulate the incident photons as a result of detector properties. The beam profile assessment algorithm adjusts the incident counts for two major detector effects according to eq. (2):

(2) $$I\left(E\right)=S_{\mathrm{EV}}^{\theta \varphi }\times D\left(E\right)\times P\left(E\right)$$

where, *I*(*E*) is photon intensity in the final form of modeled beam profile which is function of the energy component of the spectrum, *E*. *D*(*E*) is the correction coefficient for detection efficiency, and *P*(*E*) is the correction coefficient for pulse pile‐up which both are explained as follows:

Detection efficiency is one of these factors that depends on the type of sensor layer attached to the Medipix3RX (e.g., CdTe and CZT) and its thickness. The number of counts computed by the source model is corrected for the corresponding detector absorption efficiency of each MARS spectral CT by eq. (3).

(3) $$D\left(E\right)=1-e^{\mu_{E}t}$$

where, $\mu_{E}$ is linear attenuation coefficient (mm^−1^) of the sensor layer which varies with energy, *E*. The thickness of the sensor layer, *t*, which is 2 mm for the Medipix3RX detector was used in this study.

Another phenomenon which distorts the spectral performance of the detector is coincident photon pile‐up that happens when the photons arrive in a time domain less than the dead time of the detector. This results in reducing the number of recorded counts in the detector which appears in the form of a high energy tail in the x‐ray spectrum. To account for this phenomenon, the pulse pile‐up model is also applied to the source model in which a series of probability distribution functions have been defined to simulate the probability of the photon counts at 1 keV threshold steps within the energy spectrum.^22^ In this model, the absolute number of counts in the measured spectrum can be simulated according to the exposure settings, the geometry of the scanner, the pixel size of the detector, the property of the semiconductor layer, and the resolving time of the ASIC. The outputs are the correction coefficients for pulse pile‐up, *P*(*E*), which are used in eq. (2) to correct the spectrum.

### 2.B Measurement of the beam profile

**Figure 4:**
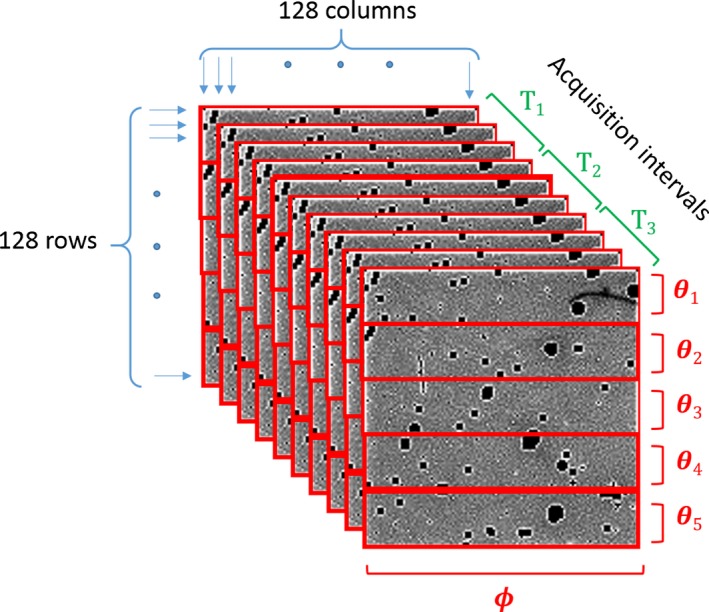
A series of flat‐field images in a single camera position taken by a CdTe‐Medipix3RX detector. The red dividers indicate how row pixels are categorized into several groups to increase the resolution of the beam profile along θ. These count groups are averaged across all frames within each time interval, labeled T_1_, T_2_, and T_3_.
- *Temporal beam profile* is measured to monitor the variation in the beam profile over time at a given position. In the small‐bore spectral CT setups with single‐chip camera, the dataset of each camera position is consecutively collected before the next one. To obtain temporal information of the recorded counts, the dataset of each detector position is classified into three acquisition intervals as shown in Fig. 4 by *T*_1_, *T*_2_ *,* and *T*_3_. Then, the beam profile of each time interval across all camera positions is constructed from the staggered time intervals throughout different positions (Fig. 5). For a segmented frame such constructed by the previous step, the mean count of each segment is then averaged across a series of frames collected in each time interval.

The spectral scanner used in this study was MARS small‐bore CT scanner equipped with a single‐chip Medipix3RX bump‐bounded to 2 mm of either CdTe or CZT. The sensitive area of this hybrid detector is 1.408 × 1.408 cm^2^ comprising 128 × 128 pixels with a pixel pitch of 110 μm. This camera is translated vertically to cover a field of view fitted to the object (i.e., five camera positions in a typical scan shown in Fig. 2). The x‐ray tube has a 20° tungsten anode with a focal spot of 50 μm manufactured by Source Ray Inc. (model: SB‐120‐350), which operates with the tube current of 10–350 μA and tube voltage of 60–120 kVp. A series of flat‐field data is acquired from the first detector channel with threshold value of 15 keV. A field of view was selected to cover the vertical translation range of θ = ± 17°. The beam profile along θ is then extracted through following steps:

- *Pixel masking* is applied to remove data from malfunctioning and poorly behaved pixels across the detector. This pixel mask is unique to each Medipix3RX detector.^23^
- *Spatial beam profile* along θ is obtained by calculating the recorded counts in the flat‐field dataset against the camera translation along θ, irrespective of acquisition time. To enhance the resolution of the beam profile, each frame is divided along θ into groups of rows and the counts across each group are averaged. In Fig. 4, each frame is divided into five groups for the demonstration purpose, but to obtain more points across the beam profile, the average of each four rows of pixels is typically used. This grouping provides 32 data points at each camera position. Thus, the beam profile resolution of a five‐camera position scan is extended up to 160 points along θ.

**Figure 5:**
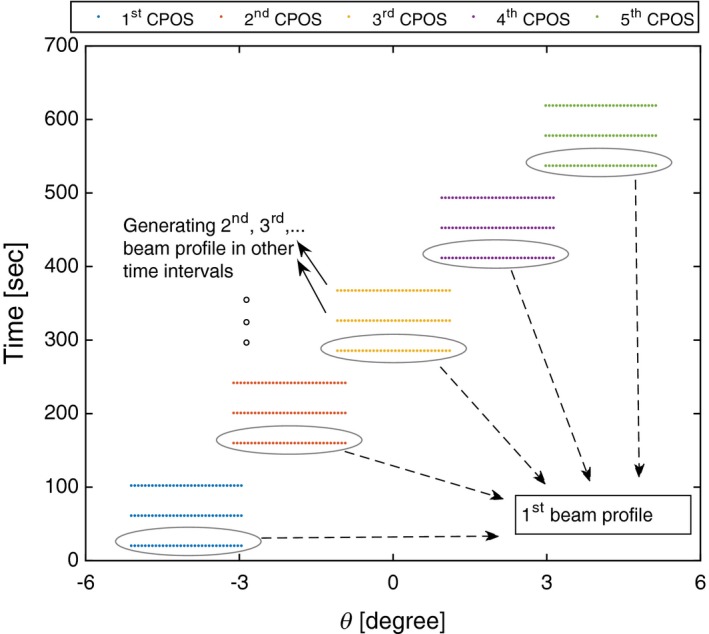
Classified counts against time and position. The measured counts classified against time and position to produce a beam profile from a scan with five camera positions (CPOS). In a MARS scanner with the single‐chip camera, the dataset of each camera position is collected sequentially. The flat‐field dataset at each camera position is divided by three, representing three time intervals (T_1_ − T_3_). This classification is shown in the graph by three horizontal dotted lines at each camera position. Each temporal beam profile is constructed by stitching data of the respective time interval from each camera position. Each dot along the horizontal lines represents the pixel classification based on the position that is calculated in the same way as illustrated by θ_1_ − θ_5_ in Fig. 3.
- *Unit conversion* is applied to the measured data in unit of “counts/pixel μA ms” to make it comparable with the modeled beam profile, which is expressed in unit of flux (counts/μsr μA ms). As shown in Fig. 2, solid angle (in yellow) is subtended by the area of the pixel as seen by the x‐ray source. The solid angle count is independent of the geometrical features of the source and planar detector alignments, such as source to pixel distance, pixel tilt, and displacement of the pixel from the beam center. By using the same unit for all measured beam profiles, this assessment approach gives the additional benefit of identifying interscan variation.
- *Regression* is applied to the measured data using a second‐degree polynomial curve fitting to extract the beam profile. This fitted curve is expected to follow the parabolic shape as the modeled beam profile. The measured beam profile obtained from this step is then normalized with respect to the peak of the parabola to assess some properties like its shape as explained in the next section.

### 2.C Beam profile properties

Several properties of the beam profile have been determined in this study to efficiently characterize the spatial beam distribution in a spectral CT scanner. The reliability of the beam profile's properties is also assessed by comparing them with properties of the modeled beam profile.

*Beam profile shapes* are assessed using the concavity and latus rectum of the beam profile parabola. To determine the sign of concavity, a simple test is to calculate the second derivative of the measured beam profile. The latus rectum of a parabola is the chord that passes through the focus, which is perpendicular to the major axis transversing the curves at two points.^24^ The measured beam profiles are expected to be concave down similar to the modeled beam profile. The average of the latus rectums in all temporal beam profiles is calculated and compared with the latus rectum of the molded beam profile. In addition, the variation in the temporal beam profiles is considered to assess the beam profile.

**Figure 6:**
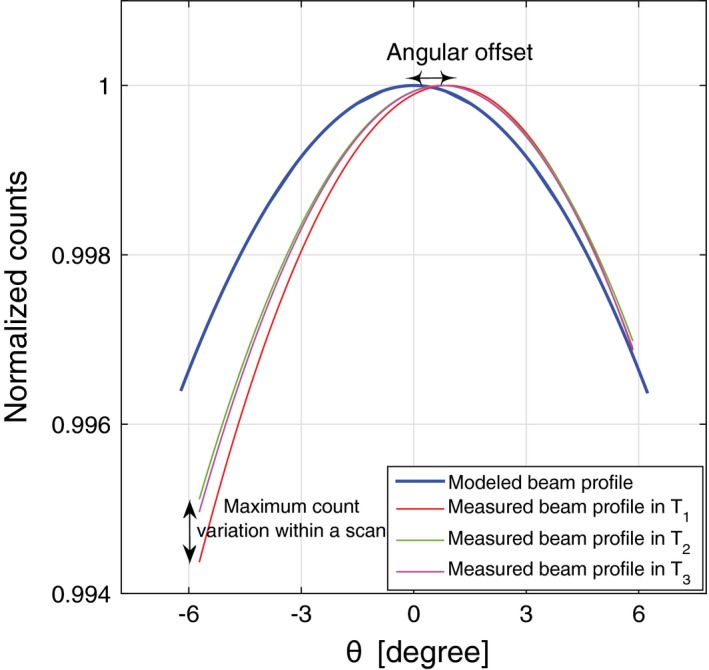
An example of quantitative assessment of the beam profile. The measured and molded beam profiles have the same concavity and small difference of the latus rectums. The arrows show how the measured beam profile parabolas deviate from the model in both properties of angular offset and intrascan variations.

Figure 6 shows a series of temporal beam profiles in a calibrated system, which are all concave down with a small variation in the latus rectums. The shapes of measured beam profiles also match the model after applying the angular offset adjustment. It is noteworthy that analyzing each of parabolic properties solely does not provide enough evidence to accurately assess the shape of the beam profile. For instance, the temporal beam profiles of a CT system with a severe anode defect may have a similar size of latus rectums, while all fitted parabolas may have the inverted concavity.

*Angular offset along* θ is the deviation of the beam profile peak from the center of rotation. An angular offset is always expected due to manufacturing tolerance of the x‐ray tube. However, the angular offset should be approximately constant with a reasonable uncertainty arising from the experimental precision (Fig. 6). The angular offset calculated for each MARS spectral CT is then used for ξ_θ_ in the source model formula [eq. (1)]. Thereby, the spectral reconstruction algorithm can also be calibrated to the actual features of the spatial photon distribution in each scanner. To conveniently compare the other properties of the measured beam profile with the model, the peaks of the measured beam profiles are adjusted to the center (θ=0).

*Intrascan count variation* is determined by calculating the maximum variation in counts between different temporal beam profiles at each position (Fig. 6). If the maximum count variation exceeds a given value, it is evidence of the occurrence of a major defect in the beam profile. In well‐calibrated systems, we have observed a subjective value of 1% of intrascan variation.

*Integrated counts at the beam center* across total acquisition time are compared with the model. It is observed that the measured integral count at the beam center matches the modeled beam profile within 1% difference in the well‐calibrated systems. The integral counts registered at each Medipix3RX counter are associated with the total number of the photons exceeds the energy threshold value set for that counter.

*Interscan count variation* can be measured by comparing count drift between different scans at the beam center. The interscan variability arises from changes in the system state such as increasing the ASIC temperature or detector polarization due to heavy use of the CT system.

### 2.D Method evaluation

To validate the efficiency of the beam profile assessment method, a MARS spectral scanner was used which completely passed a series of QA tests to check the stability of every component of the scanner such as high voltage power supply, x‐ray tube, and detector, as well as performing several geometrical alignment tests. This system, therefore, was considered as a well‐calibrated system. Three datasets including 720 flat‐field frames in each were collected by a single‐chip CdTe‐Medipix3RX at every camera position. The camera was translated to five positions and the distance from the center of the camera position to the x‐ray source was set to 187 mm. Each single exposure was performed by an 80 kVp x‐ray beam with the intensity of 30 μA during 120 ms. A 3.1 mm aluminum sheet was also used to filter the x‐ray beam in addition to an intrinsic filter of 1.8 mm aluminum.

Furthermore, the efficiency of the beam profile assessment method was evaluated by performing this method to the poorly calibrated CT systems. For these series of measurements, five camera positions were chosen and 720 flat‐field frames were collected at each position. The source to detector distance was set at 270 mm. In every flat‐field measurement, the camera was exposed by a 120 kVp x‐ray beam with the intensity of 20 μA during 180 ms. The output spectrum was filtered by 0.375 mm brass.

## 3 RESULTS

This section reports the results of the beam profile assessment method performed on several MARS spectral CTs with different levels of calibration quality.

### 3.A Beam profile assessment for a well‐calibrated system

**Figure 7:**
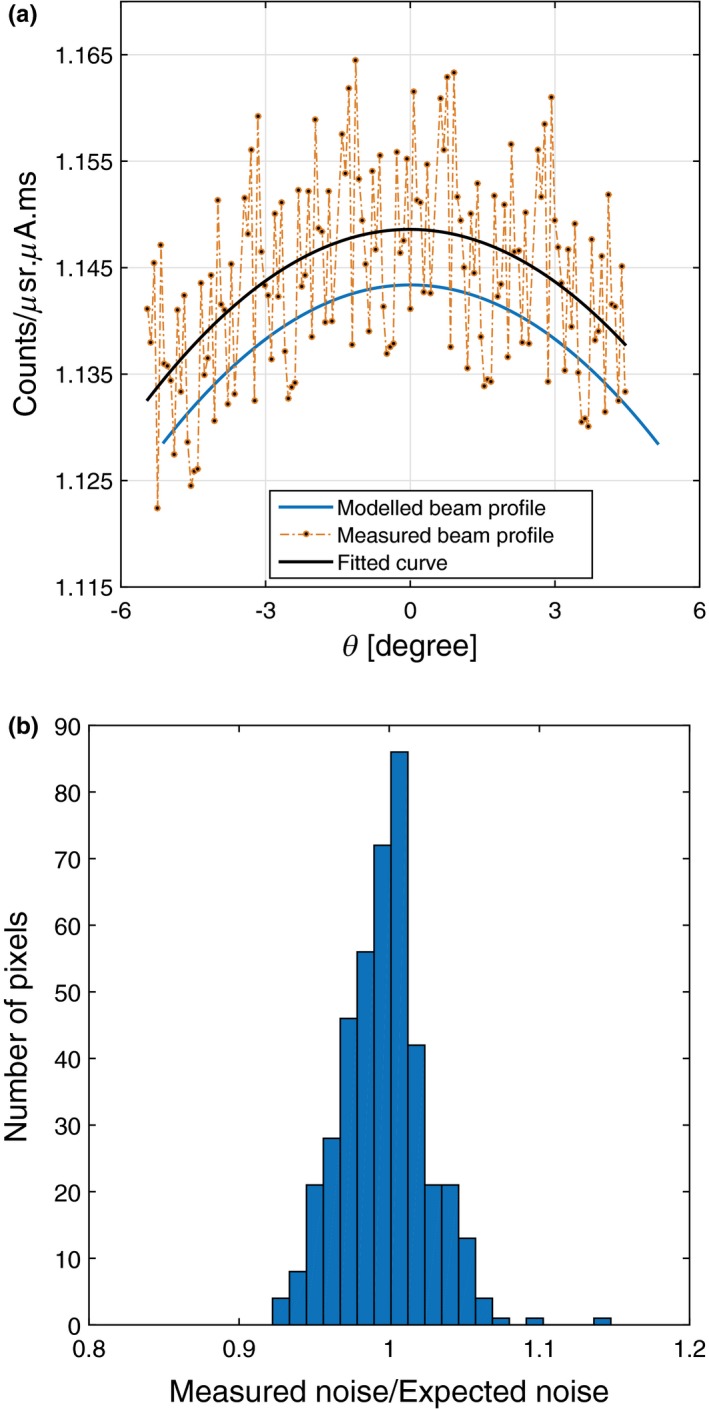
(a) The comparison of the measured counts and fitted curve plotted against the modeled beam profile. (b) Histogram of the measured to the expected noise ratio following the Poisson distribution.

The assessment beam profile method was applied to all three datasets acquired from a MARS spectral scanner considered as a well‐calibrated scanner. The photon distribution along θ from one of these datasets is demonstrated in Fig. 7a. We checked for bias in this dataset by inspecting the ratio of the measured noise (i.e., variance/mean) to the expected noise (i.e., $1/\sqrt{n}$ where n is photon flux across the number of frames for each pixel). The histogram of this ratio for a group of counts is presented in Fig. 7b. The bell‐shaped histogram with an average of one indicates a Poisson distribution. Next, a quadratic function was fitted to this dataset, as shown in Fig. 7a. The root mean square error (RMSE) of the fitted curve to this dataset is 0.0088 showing the quality of curve fitting. For two other datasets measured by this scanner, the RMSE are 0.0096 and 0.0087.

**Figure 8:**
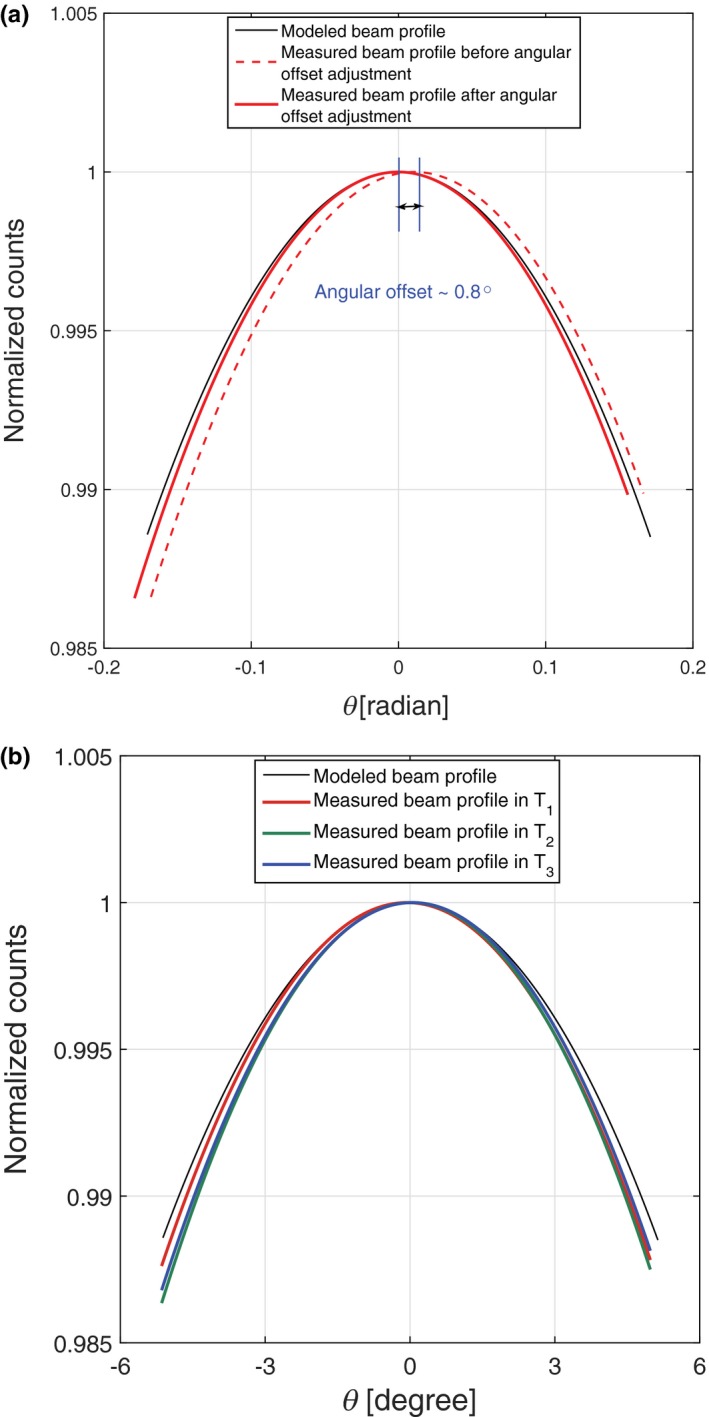
(a) Comparing the modeled beam profile shape with MARS experiments before and after angular offset adjustments. (b) Variation of the temporal beam profiles in three time intervals plotted against the modeled beam profile.

The first step of beam properties measurement is to assess the shapes of the temporal beam profiles. All temporal beam profiles are concave down with the average latus rectum of 2.26° ± 0.07°. There is 0.03° difference between the average latus rectum of the temporal beam profiles and model, which is within the experimental uncertainty. Hence, the shapes of these temporal beam profiles are well‐matched with the model as shown in Fig. 8b.

Second, the angular offset of this measurement along θ is 0.8° ± 0.07°, as shown in Fig. 8a. The solid red curve shows the measured beam profile after applying the angular offset adjustment. The standard deviation value (±0.07°) is approximately one‐tenth of the angular offset, which is low enough to accept the angular variation in the temporal beam profiles.

Third, there is an intrascan count variation in this measurement as shown in Fig. 8b. The deviation of the beam profile in the last time interval with respect to the first one is around 0.1%, which is negligible for this scan. It is evident that the beam profile is quite stable on this CT scanner.

**Figure 9:**
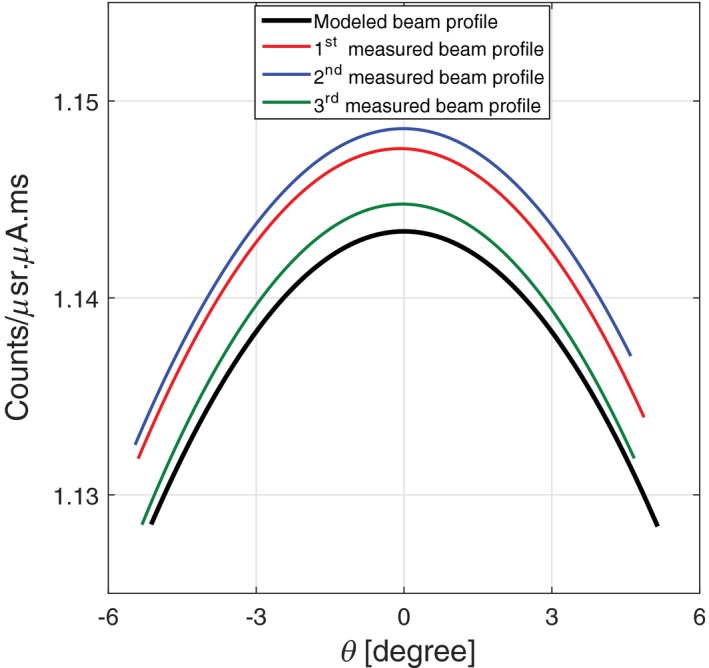
Comparison of the integrated counts between the MARS source model and three different experimental beam profiles.

Fourth, the magnitude of the measured beam profile was compared with the model at the beam center. In Fig. 9, the blue curve shows the beam profile of this dataset plotted against the model. The difference between the integral counts of this dataset and model is around 0.5% at θ=0.

Finally, the beam profiles of two other datasets collected by the same spectral CT are also plotted in Fig. 9. The interscan variation between these three datasets is just above 0.3% due to statistical error. Low interscan variation indicates that this CT system can reliably perform the same scan.

### 3.B Beam profile assessment for poorly calibrated systems

**Figure 10:**
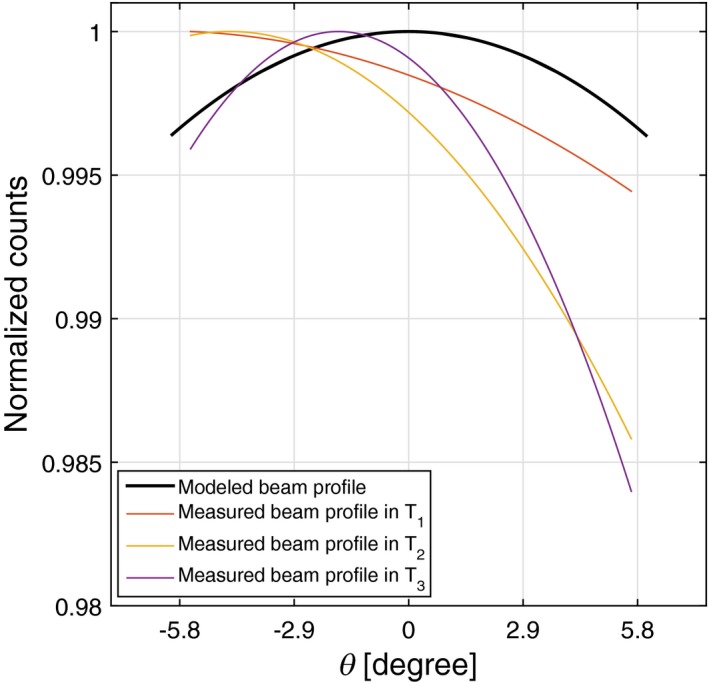
A beam profile with a minor defect due to intrascan variation at the end of the scan (i.e., most positive θ value). Although this dataset has relatively large angular offset, the variation in the angular offsets between all temporal beam profiles is within the acceptable range.

Assessment of the beam profile shapes in a poorly calibrated MARS spectral system at different time intervals indicate a minor defect in the beam profile, as shown in Fig. 10. All temporal beam profiles of this experiment are concave down with the average latus rectum of 1.02° ± 0.12°. There is, however, a large discrepancy of 1.5° between the average latus rectum of the temporal beam profiles and the model. The angular offset of this scanner is 1.6° ± 0.14°, which represented the small angular offset variation between the temporal beam profiles ranging from 1.5° to 1.8°. Count drift causes a deviation around 0.6% in the temporal beam profiles at the positive θ. The integrated count difference between the measured and modeled dataset is 25%.

**Figure 11:**
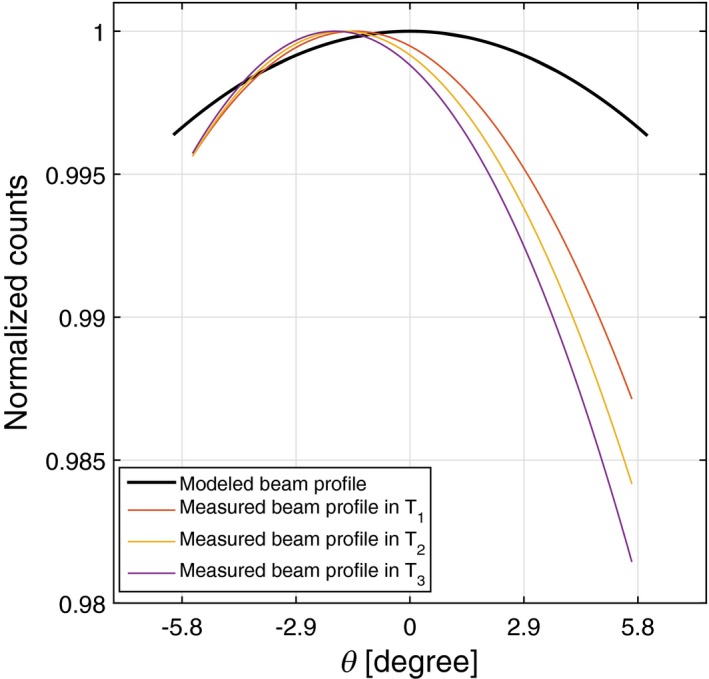
A beam profile with a major defect behaving chaotically in different time intervals. Both beam properties of intrascan variation and angular offset show large variation.

Figure 11 presents the results of the second series of measurements. As shown in this figure, the measured beam profiles have an inconsistent pattern against time and position. All of the beam profiles are concave down with the average latus rectum of 3.6° ± 3.57°. The angular offset is 3.6° ±°1.6°, which represents a large variation in angular offset between temporal beam profiles, ranging from 1.8° to 5.4°. The intrascan count variation is 1.4% and integral count difference at the beam center is 30%. On the basis of the extreme value of results, the beam profile of this system has a major defect. The beam profile properties of all experiments are summarized in Table 1.

**Table 1:** Summary of beam profile assessment results

Properties	Status		
	Stable	Minor defect	Major defect
Concavity	Concave down	Concave down	Concave down
Average latus rectum	2.26°± 0.07°	1.02°± 0.12°	3.6°± 3.57°
Latus rectum diff. from model	0.03°	1.5°	1.2°
Angular offset along (ξ_θ_)	0.8 ± 0.07°	1.6 ± 0.14°	3.6 ± 1.6°
Intrascan count variation	0.1%	0.6%	1.4%
Integral count diff. at the beam center	0.3–0.5%	25%	30%
Interscan count variation	0.3%	–	–

## 4 DISCUSSION

The results demonstrated that the beam profile assessment technique can efficiently be used to monitor the performance of the spectral CT scanner. This method could precisely exploit the parameters varying between different beam profiles.

The quadratic polynomial function fitted to the first set of experiments was evaluated by RMSE values, which was less than 1% for a series of stable beam profiles presented in this study. The results of the beam profile assessment in the MARS spectral CT with the same setup showed that even if the beam profile shape was deformed, a quadratic curve fitting could still express the actual shape of the measured profile. As a worst‐case scenario, it can be referred to an anode defect occurrence in which the beam profile may not follow the quadratic trend. It is noteworthy that the anode defect is very unlikely to happen for the low power x‐ray tubes 20 used in a spectral micro‐CT system like MARS as the amount of heat formed at the anode is a fraction of heat generated from the anode surface of the high‐power x‐ray tube used 25 in the conventional CT scanner. However, the performance of the x‐ray tube in a spectral CT needs to be tested regularly to provide ongoing assurance of the system prior to performing a scan. This test can be performed systematically using the beam profile assessment method presented by this work.

The beam profile shapes in the poorly calibrated system may suffer from transient distortion or a fixed pattern distortion, depending on the pattern of distortion and how long‐lasting that beam profile deformation is. Transient distortion does not have a repeatable pattern in the scans and mostly affects a small region and rarely the entire beam profile. If the distortion continues during the main object scan, image artifacts are very likely. Factors such as x‐ray tube output variations, bias voltage fluctuations, and inaccurate scan parameters can cause temporal faults in the recorded counts. The extreme deformation of the temporal beam profiles can be seen in Fig. 11. In fixed pattern distortion, the beam profile follows a consistent pattern during all scans in which the count level in a camera position varies from the expected value. Such a beam profile distortion can be caused as a result of inaccurate geometrical calibration of the CT scanner. The CT scanner, therefore, needs to be recalibrated for the possible geometric issues such as error in the initialization of step motors used for camera translation, misalignment between the source and detector, and error in the orientation of filter bars and collimators.

The angular offset along θ , which was measured from partially stable beam profiles that varied from 0.8° to 1.6°. As previously noted, the main reason for beam profile offset is unavoidable tolerance of the x‐ray tube during manufacturing. Another possible reason is flex of the scanner components. Unlike the angular offset variation between a series of spectral CT systems, the angular offset of the equivalent temporal beam profiles should be identical within an acceptable uncertainty. The large angular offset (Fig. 11) is another evidence of poor geometric calibration of the scanner.

The angular offset along φ was not measured in this study because the horizontal dimension of a typical field of view is small in the MARS small‐bore scanners. In the case of using a wider horizontal field of view, φ offset would need to be considered. It may change the amount of vertical angular offsets and the skewness of the beam profile that requires further investigation.

To analyze the intrascan variation in the integral counts, differences between temporal beam profiles are measured. The concept of a temporal beam profile is proposed, based on count sampling at each camera position for different time intervals. It is expected that the number of counts at each position should remain the same with reasonable uncertainty, provided that scanner components are working in a steady state during data acquisition. Therefore, any inconsistencies in these beam profiles would indicate intrascan count variation.

The location of the intrascan variation also provides some clues about the origin of variation. The deviation of the temporal beam profiles at the beginning of the scan could be due to including the x‐ray tube warm‐up time in the acquisition time. Temporal deviations that appear at the end of the scan show a degradation in recorded counts, probably resulting from a gradual rise in ASIC temperature or detector polarization during data acquisition (Fig. 10). If the beam profiles in different time domains behave chaotically (Fig. 11), it is evidence of transient distortion occurring across the entire the scan. In general, intrascan count variation can increase the variation in other beam profile properties. For instance, the large tolerance of angular offset shown in Fig. 11 is due to large intrascan count variation.

The results of assessing the integral counts at the beam center in a well‐calibrated MARS CT indicated that the measured counts and those calculated from the model are well‐agreed as shown in Fig. 7a. The minor difference (<0.5%) is due to not correcting the source model for other potential detector effects such as incomplete charge collection, cadmium fluorescence, and charge sharing.^15^, ^26^, ^27^, ^28^ In the poorly calibrated CT system, a large difference (25‐30%) was observed between the experimental and modeled counts. Possible reasons are inaccuracies in the geometric calibration, such as the source to detector distance, and filter thickness. If the scan is performed under incorrect setup parameters, detector may operates in the nonlinear dynamic range; resulting in an unstable beam profile.

The beam profile assessment in a series of scans performed by the same spectral CT scanner revealed that there is no significant interscan variation in integral counts at the beam center in a well‐calibrated system (0.3% in Fig. 9). The interscan count variation analysis can be used as a part of quality assurance (QA) test for measuring repeatability error in each spectral CT scanner. A scanner fails the assessment test when a large interscan variation is observed between the scans performed iteratively on the same day.

In human spectral CT with a larger field of view, multichip detectors are used, which require more accurate and faster troubleshooting. Using a multichip detector, the entire beam profile can potentially be acquired at a single exposure. Because of this, the overall trends of spatial and temporal beam profiles are formed by the beam profile of each individual chip in the detector array. Providing correct beam profiles for all detectors in an array is essential, particularly when they are operated in a helical scan.^17^ Translation of the beam profile assessment method from the single‐chip detector to the multichip detector array can be performed by analyzing the response of each chip. Stitching the beam profiles measured by all detectors together would provide higher resolution of the overall beam profile as more spatial points are available using the multichip detector.

Although the output of this technique indicates the beam profile fluctuation in a single‐chip camera, it does not address the main source of this fluctuation. It is expected that in a multichip camera, we can differentiate between an unstable x‐ray tube and a faulty detector array. This is because of simultaneously obtaining the correlated spectral signal in a multichip camera, over a larger θ direction by different chips. In addition, further investigation is required to precisely determine an uncertainty range for each property of the beam profile.

## 5 CONCLUSION

The method presented in this paper qualitatively and quantitatively assesses various beam profiles, which can assist in improving spectral CT performance in two ways. Firstly, the method can identify the presence of various calibration issues in a spectral CT scanner. It offers a simple and fast check of the beam profile during manufacturing. It also aids in reliably performing quality assessment at different stages from manufacturing through to the final product. Secondly, the accurate offset parameters of the beam profile provided by this work can also be used for additional geometric calibration of the x‐ray source model. The use of optimized x‐ray source model in the spectral reconstruction techniques will improve the accuracy of material identification and quantification.

## ACKNOWLEDGMENTS

The authors thank the University of Otago, University of Canterbury, MARS Bioimaging Ltd., Medipix collaborations at CERN, National Heart Foundation, and Maurice and Phyllis Paykel Trust for supporting this research.

## CONFLICT OF INTEREST

No conflicts of interest.
